# Demographic buffering: titrating the effects of birth rate and imperfect immunity on epidemic dynamics

**DOI:** 10.1098/rsif.2014.1245

**Published:** 2015-03-06

**Authors:** Sinead E. Morris, Virginia E. Pitzer, Cécile Viboud, C. Jessica E. Metcalf, Ottar N. Bjørnstad, Bryan T. Grenfell

**Affiliations:** 1 Department of Ecology and Evolutionary Biology, Princeton University, Princeton, NJ, USA; 2 Department of Epidemiology of Microbial Diseases, Yale School of Public Health, New Haven, CT, USA; 3 Fogarty International Center, National Institutes of Health, Bethesda, MD, USA; 4 Center for Infectious Disease Dynamics, Department of Entomology, Pennsylvania State University, University Park, PA, USA; 5 Center for Infectious Disease Dynamics, Department of Biology, Pennsylvania State University, University Park, PA, USA

**Keywords:** partial immunity, susceptible–infected–recovered–susceptible, acute infection, demography, birth rate, epidemiology

## Abstract

Host demography can alter the dynamics of infectious disease. In the case of perfectly immunizing infections, observations of strong sensitivity to demographic variation have been mechanistically explained through analysis of the susceptible–infected–recovered (SIR) model that assumes lifelong immunity following recovery from infection. When imperfect immunity is incorporated into this framework via the susceptible–infected–recovered–susceptible (SIRS) model, with individuals regaining full susceptibility following recovery, we show that rapid loss of immunity is predicted to buffer populations against the effects of demographic change. However, this buffering is contrary to the dependence on demography recently observed for partially immunizing infections such as rotavirus and respiratory syncytial virus. We show that this discrepancy arises from a key simplification embedded in the SIR(S) framework, namely that the potential for differential immune responses to repeat exposures is ignored. We explore the minimum additional immunological information that must be included to reflect the range of observed dependencies on demography. We show that including partial protection and lower transmission following primary infection is sufficient to capture more realistic reduced levels of buffering, in addition to changes in epidemic timing, across a range of partially and fully immunizing infections. Furthermore, our results identify key variables in this relationship, including *R*_0_.

## 1. Introduction

There is great diversity in the mechanisms by which pathogens interact with host immune systems and cause disease [1]. In the simplest cases, such as the childhood infections measles, mumps and rubella, infection is acute and a single exposure event is sufficient to confer lifelong immunity against the disease (and generally prevent further transmission) [2,3]. However, for most pathogens the outcome is complex, as immunity can wane over time or provide only partial protection against reinfection [2,4]. One of the major challenges of applying theoretical disease models to these real dynamical systems is in incorporating sufficient biological information while retaining a parsimonious modelling framework [5].

Two of the most basic mathematical frameworks are the susceptible–infected–recovered (SIR) and susceptible–infected–recovered–susceptible (SIRS) models, which have been extensively studied in the epidemiological literature and provided many insights into the underlying dynamics of infectious diseases [6,7]. The SIR model assumes immunity is lifelong and the SIRS model assumes that once immunity has waned, individuals become entirely susceptible to reinfection and, if reinfected, will be equally infectious as an individual exposed for the first time. These simple frameworks have been applied to a wide range of pathogens, from perfectly immunizing infections such as measles and rubella in the case of the SIR framework, to imperfectly immunizing infections such as influenza and syphilis in the case of the SIRS [7–10]. However, the assumptions of these standard models represent two extremes of the spectrum of possible immunological dynamics within the host and do not capture the biology of many infectious diseases [4,7].

For many imperfectly immunizing pathogens, primary infection has been shown to have a significant impact on the outcome of subsequent host exposures to infection. In the case of rotavirus, respiratory syncytial virus (RSV) and *Bordetella pertussis* (pertussis), previously exposed individuals are partially protected against reinfection, and those individuals that are reinfected may be less infectious and experience reduced severity of disease [11–16]. Consequently, variations on the standard SIR(S) framework have been applied in certain contexts, for example with the incorporation of waning of immunity and immunological boosting following re-exposure to pertussis [17,18]; the distinction between primary, secondary and asymptomatic rotavirus infections [19]; and the inclusion of partial immunity to RSV reinfection [20]. Accounting for different immune dynamics can increase the biological realism of the standard models and enhance our ability to capture and understand the observed epidemiological patterns of real systems.

A key probe for understanding infectious disease dynamics is the impact of demography on population-level patterns of incidence [21,22]. In the case of immunizing infections, long-term changes in demographic parameters of the host community, such as birth and immigration rates, can have a direct impact on the recruitment rate of susceptible individuals and thus on the overall infection dynamics. In particular, disease dynamics have been shown to be highly sensitive to varying birth rates, as models allowing changes in birth rate and vaccination levels can account for the complex switching from annual epidemics to irregular and multiennial cycles in measles incidence [22]. By contrast, the loss of immunity of previously infected individuals provides an additional source of susceptibles for imperfectly immunizing infections that can, in turn, lead to more frequent epidemics [2]. This can be seen in the dynamics of influenza A, where annual epidemics are fuelled by the rapid replenishment of susceptible individuals as novel strains evade the acquired immunity of previously infected hosts [23,24]. However, the sensitivity of such systems to demographic changes, in particular, the role of imperfect immunity in modulating the response to birth rate variations, is poorly understood.

Here we use the term ‘buffering’ as a measure of the sensitivity of disease dynamics to changes in demographic parameters: if demographic changes have a negligible impact on the patterns of disease spread, there is high buffering (or the host population has been ‘buffered’ against the effects of demographic variation), whereas low buffering arises when disease dynamics and demographic changes are highly correlated. We first analyse the buffering patterns predicted by the SIR(S) framework and show that as the duration of immunity decreases, these standard models predict that disease dynamics should become less sensitive to variations in birth rate, so that the level of buffering increases. We then compare these predictions to observed epidemic dynamics and find discrepancies for a number of partially immunizing infections, suggesting that an alternative framework is necessary. To address this discrepancy, we introduce a refined mathematical framework that provides a link between the SIR and SIRS models and accounts for the waning of host immunity, and reduced susceptibility and infectiousness following primary exposure to a pathogen. We show that this modified model predicts a range of buffering patterns that are not only qualitatively different from those of the standard models, but also better capture the patterns observed in reality for a number of acute infections.

## 2. Preliminary model and analyses

### 2.1. SIR(S) model

The SIRS model with constant population size is given by equations (2.1) [7,25], where *S* denotes the number of susceptible individuals, *I* the number infected, *R* the number immune and *N* the total population2.1
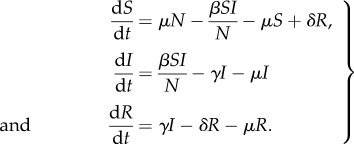


We define *μ* as the birth (and death) rate, *β* the effective transmission rate, *γ* the rate of recovery from infection and *δ* the rate at which individuals lose immunity. The SIR model is a special case of the SIRS model in which *δ* = 0 (i.e. individuals never lose immunity). The term *βI*/*N* represents frequency-dependent transmission, which assumes the rate of infection is independent of population density [25]. For a constant population, this is equivalent to density-dependent transmission, which alternately assumes infection rate scales with population density [25]. However, when the population size is changing these two frameworks are no longer equivalent, and we consider such cases in our simulations below. A summary of all parameters, and the values assigned for these parameters in simulations, are provided in table 1.

**Table 1.** Model parameters: values of all parameters used in simulations for the standard SIR(S) and the modified model, unless stated otherwise. Ranges indicate parameters that are varied across simulations as part of model analyses.

parameter	symbol	value
reproductive number	*R*_0_	10–25
recovery rate	*γ*	1/4 week^−1^
population size	*N*	10 000
birth rate	*μ*	0.01–0.02 yr^−1^
rate of loss of immunity	*δ*	0–1 yr^−1^
average transmission rate	*β*_0_	(*μ* + *γ*)*R*_0_
seasonal transmission rate	*β*	*β*_0_(1 + 0.175 cos(2*πt*))^a^
relative infectiousness of subsequent infections	*α*	0–1
relative susceptibility of subsequent infections	*ε*	0–1
proportion of subsequent infections that are reported	*r*	0–1

^a^Time is represented by the variable *t* and is measured in years so that the function governing the seasonal transmission rate has annual periodicity.

If we assume the infection is acute so that 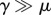, then the endemic equilibrium of infected individuals (I^*^ ≠ 0) [6] is2.2
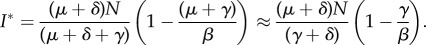


The basic reproductive number, *R*_0_ = *β*/(*γ* + *μ*), is the average number of secondary infections generated by an infectious individual in an entirely susceptible population [5,25]. For a given value of *R*_0_, the proportion infected at equilibrium increases with increasing *δ*, whereas the proportion susceptible remains essentially unchanged (electronic supplementary material, figure S1).

### 2.2. Theoretical analysis

Using equation (2.2), we explore the impact of a change in birth rate on disease incidence. Suppose the new birth rate is given by 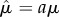, then the new endemic equilibrium is
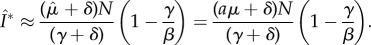


And the proportional change in equilibrium disease incidence induced by this demographic change is2.3
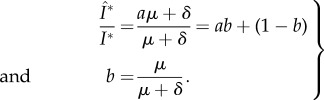


The change in birth rate is 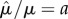. Hence, *b* is the slope of the regression line of change in birth rate against change in disease incidence (equation (2.3)) and indicates the extent of buffering resulting from changes in the population demographics. For example, if *δ* = 0, then *b* = 1 and Î^*^/I^*^ = *a*, and so the SIR model predicts that a change in birth rate will lead to a proportional change in disease incidence. However, as *δ* → ∞, *b* → 0 and Î^*^/I^*^ → 1. Thus, the SIRS model predicts that increasing the rate of loss of immunity will buffer the population against changes in disease incidence that would otherwise be induced by changes in the population birth rate.

### 2.3. Simulations

**Figure 1.**
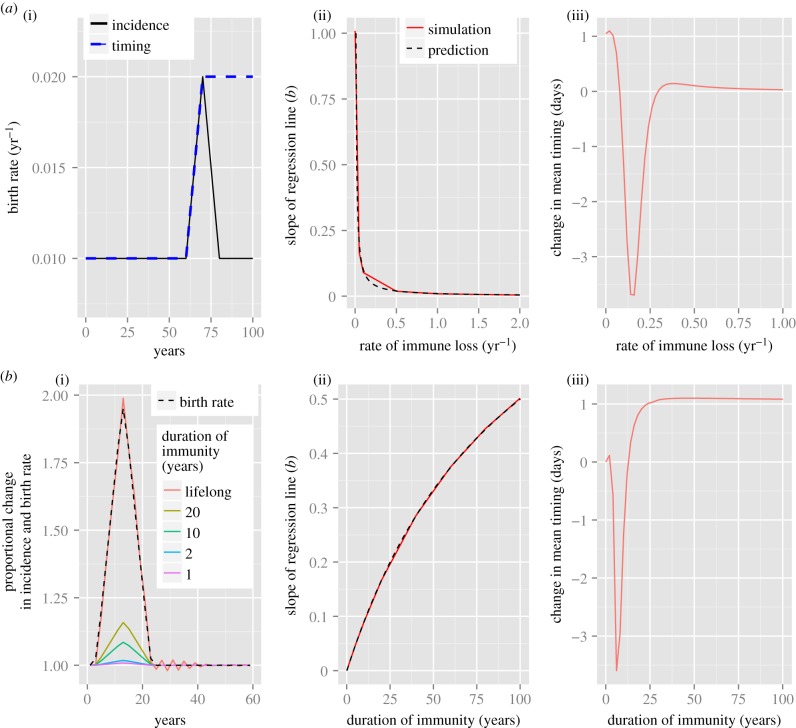
(*a*(i)) Functional forms of the birth rate used to investigate the extent of buffering in relation to changes in disease incidence (black solid line) and epidemic timing (dashed blue line) for both the standard and modified models. (*b*(i)) Comparing the proportional change in incidence for the SIR (lifelong immunity) and SIRS models with different durations of immunity in response to a change in birth rate. The plotted results represent the proportional changes in incidence (solid lines) and birth rate (dashed line) for the period immediately following the initiation of the birth pulse. (*a*(ii), *b*(ii)) Plotting the slope of the regression line of change in birth rate against change in disease incidence gives a measure of the extent of buffering in response to demographic variation. The red line is the result of simulations for the SIRS model (where *δ* = 0 corresponds to the SIR model) and the dashed black line corresponds to the theoretical prediction, *b* = *μ*/(*μ* + *δ*). Results are plotted as a function of rate of loss of immunity (*a*) and duration of immunity (*b*). (*a*(iii), *b*(iii)) Change in mean timing (days) induced by a linear increase in birth rate. The change is the difference between the mean day of the epidemic during the final year of the simulation and the year immediately prior to the change in birth rate (as discussed in §2). Again, results are plotted as a function of rate of loss of immunity (*a*) and duration of immunity (*b*). The reproductive number, *R*_0_, is 17 and all other parameters for the SIRS model are as defined in table 1.

We simulate the effect of a change in birth rate on seasonally forced SIR(S) dynamics for a range of values of loss of immunity (figure 1). The resulting buffering levels with respect to disease incidence are compared using the measure *b* (equation (2.3)), and the change in timing of seasonal epidemics are compared using two measures: (i) the mean timing, or centre of mass, of the epidemic and (ii) the timing of the peak in number of infected individuals (see the electronic supplementary material, §S2, for further details). The functions representing the changing birth rate are depicted in figure 1*a*(i) and all parameter values are given in table 1.

As predicted above, there is no buffering when *δ* = 0. However, as the rate of loss of immunity increases, there is a sharp decrease in the slope of the regression line indicating an increase in the extent of buffering (figure 1*a*(ii)). Alternatively, the average duration of immunity is 1/*δ*, and so the extent of buffering decreases as the duration of immunity increases (figure 1*b*(ii)). Moreover, the analytic prediction of equation (2.3) and the simulated results in figure 1 are in extremely close agreement, and this agreement is robust to the following changes to the model: (i) relaxing the constraint of constant population size (i.e. allowing different birth and death rates), (ii) defining transmission as density-dependent instead of frequency-dependent, and (iii) assuming ‘relapse’ dynamics as in the SIRI model [2], where individuals move straight into the infected class after losing immunity (results not shown). Thus, the phenomenon of buffering is consistently predicted across a range of variations of these standard models, and equally at equilibrium (as in the theoretical analysis) or with seasonally forced temporal dynamics (as in the simulations).

The change in mean timing of the epidemic in response to increasing birth rates also depends on the rate of loss of immunity (figure 1*a*(iii), *b*(iii)). The greatest decreases in mean timing of the seasonal oscillations (indicating earlier epidemics) occur at intermediate durations of immunity. Otherwise, there is no change in mean timing for shorter durations of immunity and a slight increase for longer durations of immunity. The SIRS model thus predicts that for diseases with high rates of loss of immunity, a change in birth rate will have negligible impact on the timing of epidemics, indicating another form of buffering. Using the change in peak timing as an alternative measurement gave qualitatively similar results, although the model predictions had a larger range of magnitude (results not shown).

### 2.4. Matching observed patterns

**Figure 2.**
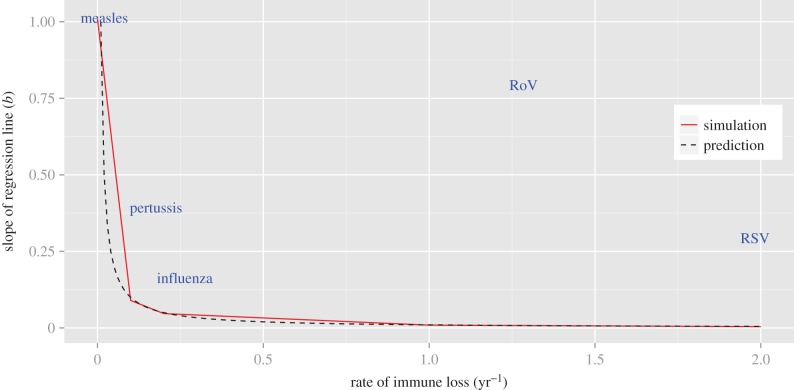
Plotting the slope of the regression line of change in birth rate against change in disease incidence gives a measure of the extent of buffering in response to demographic variation. The red line is the result of simulations for the SIRS model (where *δ* = 0 corresponds to the SIR model) and the dashed black line corresponds to the theoretical prediction, *b* = *μ*/(*μ* + *δ*). Results are plotted as a function of rate of loss of immunity and approximate buffering regions where we expect particular pathogens to lie are indicated by the name of each pathogen (‘RoV’ stands for rotavirus). The birth rate function is as shown in figure 1*a*(i); the reproductive number, *R*_0_, is 17, and all other parameters for the SIRS model are as defined in table 1. Pathogen-specific parameters are as defined in the electronic supplementary material, table S1.

The SIR(S) framework predicts that the dynamics of pathogens conferring protective immunity for up to a decade will remain relatively unaffected in the face of substantial demographic change. In other words, strong buffering should manifest above a relatively low threshold for the rate of loss of immunity. Comparing these predictions to our current understanding for particular pathogens suggests that although the SIR(S) model may sufficiently capture patterns for diseases such as measles, influenza and pertussis, it is inadequate for partially immunizing infections such as rotavirus and RSV (figure 2, see the electronic supplementary material, table S1, for pathogen-specific parameters).

#### 2.4.1. Comparing observations to model predictions

Measles dynamics have indeed been shown to be highly sensitive to changes in population birth and vaccination rates [22], and previous work has demonstrated a significant but weak correlation between birth rate and pertussis dynamics that supports model predictions of moderate to high buffering [21]. Studies explicitly linking influenza dynamics to population birth rates are lacking; however, rich data from France and The Netherlands spanning the last 30 years suggests incidence has remained relatively stable despite both increases and decreases in population birth rates [26–28]. Thus, although demographic factors such as the age structure of a population are thought to be important modulators of influenza dynamics [29], model predictions of high buffering in response to birth rate fluctuations may still be a reasonable prediction.

By contrast, recent work suggests that partially immunizing infections such as rotavirus and RSV may be more sensitive to population birth rates than predicted by the SIRS model. It has been found that decreasing birth rates in certain US states can account for rotavirus outbreaks appearing later in the year [19]. This contradicts high buffering predictions since the duration of immunity to reinfection with rotavirus is short-lived (typically less than 1 year) [30]. Also, a shift in seasonality and declining incidence of rotavirus cases in Japan from 1985 to 1990 coincided with significant decreases in the population birth rate that could not be explained by climatic factors or the prevalent virus serotype [31,32]. Although no causal relationship between birth rate and disease incidence has been established, this supports the idea that variations in demographic parameters may be more significant than the standard SIRS model predicts. In the case of RSV, another pathogen with short-lived immunity [33], an increase in birth rates in Sweden from 1983 to 1991 was followed by a reported increase in detected RSV cases in Stockholm from 1984 to 1992 [34]. In addition, recent modelling work suggests a transition from biennial RSV epidemics in California in the 1990s to annual epidemics in the 2000s may be explained by changes in the state birth rate, although further work is needed to understand these patterns [35]. Again, although no formal connection has been established, these observations are inconsistent with the high level of buffering predicted by the SIRS model.

#### 2.4.2. Comparing observations to model assumptions

The SIR and SIRS models assume that after recovering from infection, individuals have either lifelong immunity or are only temporarily protected, and after losing that protection will become entirely susceptible again. However, the dynamics of immunity for many pathogens do not fit these two extreme scenarios, and may be better described as lying somewhere on a continuous spectrum between the SIR and SIRS frameworks. In the case of rotavirus, previous work has shown that individuals experience a relative risk of 34–62% of becoming reinfected following one or more previous exposures, and will be less infectious and experience reduced severity of disease upon reinfection [11–13]. Similarly, a relative reinfection risk of 44–75% has been estimated in the case of RSV, again with milder disease symptoms expressed during subsequent exposures [14,15,36,37].

The recent resurgence of pertussis has also stimulated great interest in the nature of host immunity in response to pathogen infection [38]. Estimates for the duration of natural immunity range between 7 and 20 years and re-exposures have also been associated with reduced infectiousness and severity of infection [16,39,40]. By contrast, although infection with influenza A virus can provide lifelong protection against the infecting strain and even partial protection against other antigenically similar strains within the same subtype, individuals can also rapidly regain susceptibility as novel strains are introduced into the population via high rates of viral mutation (i.e. antigenic drift) [24,41–43]. For example, one study found that 90% of tested individuals infected with influenza A H1N1 in 1978 were protected against reinfection the following year, but by 1983 the number protected against clinical disease dropped to 55% [44]. We base our parameters on this previous study in order to include influenza in our comparisons, but in general there is much work still to be done on understanding the interaction between host immunity and strain evolution for this particular pathogen.

#### 2.4.3. Connecting assumptions and predictions

Although the different types of immunity outlined above are defined by processes at the individual level, they can have a profound impact on the patterns of infection seen at the population level, and a greater understanding of the dynamics emerging from these underlying mechanisms is needed. In particular, why does the SIR(S) framework capture observed buffering patterns relatively well for some partially immunizing infections (such as pertussis and influenza) but not for others (such as rotavirus and RSV)? And what additional biological processes must be included in the model to improve predictions for these latter pathogens? We now introduce a refined version of the SIRS model that incorporates a greater range of the spectrum of possible host immune responses by accounting for reduced susceptibility and infectiousness following primary exposure to the pathogen. We show that our mathematical framework can capture the variation in buffering dynamics observed across this range of different pathogens, and explore the relative importance of the additional immune components in achieving this better representation.

## 3. Modified model

Here, we present a general framework that models partial immunity by allowing individual infectiousness and susceptibility to change with infection status. We investigate the range of dynamics that can be captured by varying the parameters describing the nature of individual partial immunity and population-level demographics. Our framework is based on previous formulations of more specific models, for example, those developed for capturing rotavirus and RSV dynamics [19,20], and is given by the below equations, where the subscripts P and S represent primary and subsequent infections, respectively,3.1
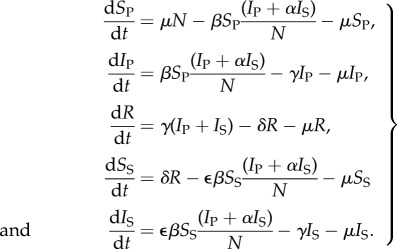


These equations reflect two biological refinements compared to the standard SIRS equations: (i) transmission in the subsequent classes is now reduced by a factor *ε* to account for a reduced risk of reinfection (so the *S*_S_ class can effectively be thought of as a partially immune class) and (ii) the contribution of individuals in the *I*_S_ class to the overall force of infection is reduced by a factor *α* to account for reduced infectiousness. The SIRS model is a special case of this general model with *ε*,*α* = 1, whereas *ε*,*α* = 0 corresponds to the SIR model. Varying the parameters *ε* and *α* between 0 and 1 thus provides a continuous link between these two standard models along the spectrums of host susceptibility and infectiousness. Similar models incorporating reduced susceptibility and infectiousness following primary infections have also been developed for pertussis and typhoid [18,45].

## 4. Results

Similar to §2.3, we simulate the effect of a change in birth rate on seasonally forced disease dynamics for a range of values of duration of immunity and reduced susceptibility and infectiousness following primary infection. In addition to comparing the resulting levels of buffering with respect to disease incidence and changes in epidemic timing, we also explore the impact of *R*_0_ and the reporting levels of subsequent infections on the model predictions. The functions representing the changing birth rate are depicted in figure 1*a*(i) and all parameter values are given in table 1 (see §2 for further details).

### 4.1. Buffering of disease incidence

**Figure 3.**
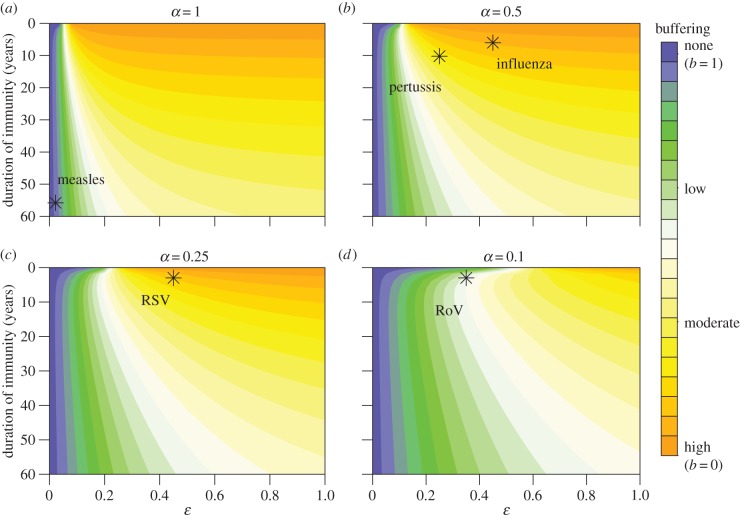
Using the slope of the regression line of birth rate against disease incidence to measure the extent of buffering in response to a change in birth rate for the modified model. Slopes close to 0 correspond to high buffering and slopes near 1 correspond to low or no buffering. The duration of immunity is 1/*δ*, where 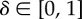, and *ɛ* and *α* are the relative susceptibility and infectiousness of subsequent infections, respectively. The birth rate function is as shown in figure 1*a*(i); the reproductive number, *R*_0_, is 17 and all other parameters for the modified model are as defined in table 1. Points represent regions of expected buffering levels for the different labelled diseases (‘RoV’ stands for rotavirus), and pathogen-specific parameters are as defined in the electronic supplementary material, table S1.

The extent of buffering of disease incidence in the modified model decreases with increasing duration of immunity (1/*δ*), and decreasing susceptibility (*ε*) and infectiousness (*α*) following primary infection (figure 3). When relative susceptibility is varied continuously between 0 and 1, and relative infectiousness is fixed (at 0.1, 0.25, 0.5 and 1), the full range of buffering levels is predicted across the region of parameter space explored (i.e. *b* ranges from 0 to 1). By contrast, varying relative infectiousness continuously between 0 and 1, at fixed values of relative susceptibility (0.1, 0.25, 0.5 and 1), does not produce the same amount of variation (electronic supplementary material, figure S2). This suggests that buffering patterns are more sensitive to changes in relative susceptibility than infectiousness.

In addition, the buffering dynamics are less sensitive to changes in the duration of immunity when there is a large reduction in relative infectiousness or susceptibility (i.e. when *α* or *ε* are small). For example, when *α* = 1 and *ε* > 0.2, the changes in buffering are largely due to changes in duration of immunity (figure 3*a*). In fact, the buffering levels across this region remain comparable to those predicted by the SIRS model at the boundary, where *ε* = 1. However, when the relative susceptibility is below this threshold, changes in buffering appear more correlated with changing susceptibility than duration of immunity, and there is a significant deviation from the SIRS model predictions. The threshold value of relative susceptibility at which this qualitative change occurs also increases as relative infectiousness decreases, such that deviations from the SIRS model become more apparent (figure 3*b*–*d*).

We can predict buffering levels for a particular pathogen using estimates for the duration of immunity, and relative susceptibility and infectiousness following primary infection. As an example, projected regions of the parameter space corresponding to rotavirus, pertussis, RSV, influenza and measles are shown in figure 3 (estimates for *α*, *ε* and *δ* are given in the electronic supplementary material, table S1). The modified model predicts high levels of buffering for influenza, moderate to high for pertussis and RSV and low for rotavirus, in line with the biology of these respective pathogens. This contrasts with the SIRS predictions of high buffering for the latter two pathogens.

In the case of the models with changing population size, the qualitative relationship between the parameters describing partial immunity and the extent of buffering are similar to that of the model with constant population size. Higher levels of buffering are seen in the frequency-dependent model (electronic supplementary material, figure S3) than in the density-dependent model (electronic supplementary material, figure S4). However, this result is unsurprising as transmission rates scale with population size in the density-dependent model and so we would expect greater sensitivity to demographic changes as a result.

#### 4.1.1. Effects of the level of case reporting and *R*_0_

An additional degree of freedom can be incorporated by varying the proportion of subsequent infections that are observed. In the case of rotavirus and RSV, this captures the fact that subsequent cases may be under-reported due to reduced severity of infection [11,37]. This is included in the modified model by defining the total number of observed infections as *I*_P_ + *rI*_S_, where 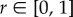 is the proportion of subsequent cases that are reported. As *r* decreases, the level of buffering also decreases across given values of *ε*, *δ* and *α* (electronic supplementary material, figure S5). Thus, incorporating *r* < 1 into the model produces larger regions of parameter space in which observed incidence closely tracks the birth pulse and in which the predictions of the modified model differ from those of the SIRS model.

The SIRS model also predicts that, for any given duration of immunity, the level of buffering does not change perceptively with variations in *R*_0_ (*ε* = 1 in electronic supplementary material, figure S6). However, for a given value of *ε* < 1, the modified model predicts that buffering increases with increasing *R*_0_ (electronic supplementary material, figure S6). Moreover, as *ε* decreases towards 0, this sensitivity of buffering to variations in *R*_0_ becomes greater. Decreasing *α* instead of *ε* produces similar patterns, although again there is less variation in the range of predicted buffering levels (electronic supplementary material, figure S7).

### 4.2. Buffering and epidemic timing

**Figure 4.**
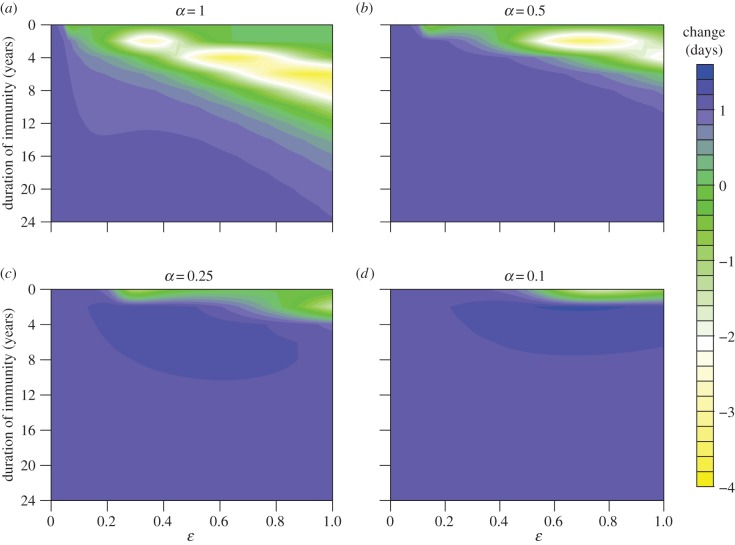
Change in mean timing (days) of epidemic oscillations induced by an increase in birth rate from 0.01 to 0.02 yr^−1^ as defined previously. Negative values indicate epidemics appeared earlier. The duration of immunity is 1/*δ*, where 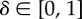, and *ɛ* and *α* are the relative susceptibility and infectiousness of subsequent infections, respectively. The birth rate function is as shown in figure 1*a*(i); the reproductive number, *R*_0_, is 17, the proportion of subsequent infections that are reported, *r*, is 1 and all other parameters are as defined in table 1.

Finally, assuming all cases are reported (*r* = 1) and considering the effect of changing birth rate on the timing of epidemics demonstrates that as relative susceptibility (*ε*) decreases, the greatest shifts in mean timing (in absolute value) occur at shorter durations of immunity compared with the SIRS model (figure 4*a*). Furthermore, decreasing relative infectiousness (*α*) leads to a contraction in the region of parameter space over which these greater changes are observed (figure 4*b*–*d*). Changing the value of *r* does not have a significant effect on these overall patterns (electronic supplementary material, figure S8), and using the timing of the epidemic peaks as an alternative measurement also reveals similar qualitative dynamics (electronic supplementary material, figure S9). Although the magnitude of the predicted changes is relatively small, the density-dependent model with changing population size does show significantly greater changes in timing (electronic supplementary material, figures S10 and S11) than both the constant population model and the frequency-dependent model (electronic supplementary material, figures S12 and S13), again likely due to the greater sensitivity of the latter to changes in population size (see the electronic supplementary material, §S4.3, for further details).

## 5. Discussion

The underlying demography of a population has long been recognized as having an influence on the spread of infectious diseases [46–49]. Typically, changes in the population birth and death rates cause the demographic structure to change over long time scales, and so it is important to understand how variations in these parameters will affect the long-term dynamics of disease. In addition, vaccination of infants at birth tunes susceptible recruitment and is thus dynamically comparable to a reduction in the population birth rate [19]. Previous studies have shown that changes in birth and vaccination rates can have a significant impact on the epidemic patterns of perfectly immunizing infections [22,50,51], but the effect on imperfectly immunizing infections is less well understood. Thus, exploring the interactions of individual-level immunity parameters and demographic factors, and the comparative impact of imperfectly immunizing vaccination, could yield important insights into the drivers of epidemic patterns observed at the population level.

### 5.1. Matching observed patterns

We have shown that the SIRS model predicts the extent of buffering will increase as the duration of immunity decreases, with almost no change in disease incidence for pathogens with short durations of immunity such as rotavirus and RSV. However, changes in birth rates in Japan, Sweden and the USA have coincided with a notable change in incidence for both of these pathogens that has not been definitively explained by variations in other extrinsic variables [32,34,35]. In contrast to the SIRS framework, the modified model predicts lower levels of buffering for both rotavirus and RSV, and thus better captures the patterns observed in reality.

We also demonstrated that the SIRS model predicts changes in birth rates and will have a negligible effect on the timing of epidemics for pathogens conferring particularly short durations of immunity. However, Pitzer *et al.* [19] found that variation in birth rates in the USA during 1990–2006 caused differences of up to three months in the mean timing of rotavirus epidemics. This study assumed that transmission was density-dependent, an assumption that has been used in other rotavirus models [52,53] and is commonly recognized for directly transmitted pathogens [54,55]. Although the results from our modified model with constant population size do not fit the patterns observed in this study, our density-dependent model with changing population size does predict greater changes in timing that are in closer agreement with the observed rotavirus dynamics than the standard models.

It is also clear that the SIRS framework can sufficiently capture observed patterns for influenza and pertussis, because these pathogens lie above the threshold of relative susceptibility and infectiousness that indicates when buffering levels are more sensitive to changes in duration of immunity than relative infectiousness or susceptibility. In other words, the reduced susceptibility and infectiousness following primary exposure to these pathogens are not significant enough to strongly deviate buffering dynamics away from those of the SIRS model.

### 5.2. Mechanisms driving model predictions

In contrast to the standard framework, our modified model allows a greater range of biologically relevant system dynamics to be modelled through tuning of the additional parameters describing partial immunity (*ε*, *α* and *r*). Recruitment into the primary susceptible class occurs via births into the population, whereas recruitment into the subsequent susceptible class results from the loss of immunity of individuals recovered from either primary or subsequent infections. This replenishment of the subsequent classes is one of the main drivers of the buffering dynamics. The greater the replenishment of susceptibles into the subsequent class relative to that of the primary class, the less impact we would expect changing birth rates to have on the epidemic dynamics, and hence the more buffering we would expect to see. For a given duration of immunity, however, decreases in the relative susceptibility and infectiousness of reinfections lead to a reduction in the proportion of individuals in both infectious classes. Consequently, the rate of recruitment into the subsequent susceptible class is reduced and so the level of buffering decreases. Furthermore, if only a proportion of subsequent infections are reported then the observed ratio of primary to subsequent cases will be greater than the actual ratio, also causing the observed dynamics to appear more sensitive to changes in the birth rate. Thus, for any given duration of immunity, by distinguishing between primary and subsequent infections, the rate at which individuals enter these classes, and the level at which infections in these classes are reported, the modified model can capture a range of different buffering scenarios that the SIRS model cannot.

In general, the greatest changes in epidemic timing are seen in the regions of parameter space associated with moderate to high levels of buffering, which is likely to be due to a complex interaction between competing elements of the system dynamics. On the one hand, higher levels of buffering indicate less sensitivity of the system to changes in the birth rate and suggest changes in the timing of epidemics should be less likely. However, greater levels of buffering are correlated with higher values of *δ*, *ε* and *α*, which in turn are associated with higher proportions of the population infected at any given time. Although increasing the rate of primary susceptible recruitment will have less of an impact on the proportional change in the number of new infections occurring in this region of parameter space, it may still impact the rate at which new infections occur and thus have a more significant effect on the timing of the epidemic oscillations.

Another important epidemiological measure is the reproductive number, *R*_0_, which encapsulates key characteristics of a particular pathogen such as the infectious period and infectiousness of an infected host [5]. Estimated *R*_0_ values can vary across geographical regions. For example, the range of *R*_0_ estimates for measles include 12.5 in North America, 13.7–18 in England and Wales, and 4.7–15.7 in Niamey, Niger [56,57]. The SIRS model predicts that buffering does not vary with changing *R*_0_, whereas the modified model demonstrates a positive relationship between these two quantities. Increasing the value of *R*_0_ is equivalent to increasing the rate at which individuals become infected. Thus, for a given duration of immunity, higher *R*_0_ values lead to more rapid transition of individuals into the subsequent classes, thereby resulting in more buffering as described above. Areas with higher estimated *R*_0_ may therefore be prone to higher levels of buffering than those with lower *R*_0_, contributing to regional differences in sensitivity to demographic changes that would not be captured by the standard models [58]. However, more studies comparing the relationship between patterns of disease incidence and changes in population demography across different communities are needed to investigate this idea further.

### 5.3. Caveats and future directions

The need for more data to explore and validate the predictions of our general framework is one of the main caveats to this study. Although we have shown that the modified model can capture patterns of buffering observed for rotavirus in Japan and RSV in Sweden, future modelling work is needed to establish a definite link between the changes in birth rates and disease incidence recorded in these regions [32,34]. Furthermore, a more mechanistic understanding of the sensitivity of pertussis and influenza dynamics to underlying population demographics, similar to previous analysis of the SIR model [22], is needed to fully explain the importance of immunological dynamics in shaping emerging epidemic patterns.

#### 5.3.1. Data

In general, more data and studies investigating the relationship between extrinsic demographic parameters and population-level disease dynamics are needed to verify our findings across a wider range of pathogens and host communities. Particular time periods of interest are those that coincide with substantial demographic change, such as the post-World War II ‘baby boom’ era in the UK [51,59] and the periods of the so-called ‘demographic transition’ that a number of developing countries have undergone in the past several decades [60]. Another period of significant birth rate variation, which caused a shift in the spatial pattern of annual rotavirus epidemics, has occurred across the USA over the past 20 years [19]. It would be interesting to explore in greater detail the relative impact of this demographic change on the dynamics of other imperfectly immunizing infections such as influenza and RSV, and test the predictions of our general model within a spatial context.

Other valuable time series may be those that span the introduction of mass vaccination campaigns for imperfectly immunizing childhood diseases, such as rotavirus and pertussis, for which vaccination may provide only partial immunity [38,61–63]. Defining the success of a vaccination campaign as the proportional reduction in disease incidence following the implementation of the campaign is thus equivalent to describing the extent of buffering in response to a reduction in susceptible recruitment rate. This could provide an additional opportunity to test our model and explore how imperfect immunity conferred by vaccination may contribute to the strength of buffering, and thus the overall success of various immunization programmes. Our prediction that buffering levels are more sensitive to changes in relative susceptibility of reinfections rather than infectiousness could also be explored further in relation to the question of which immune mechanisms should be targeted by imperfect vaccines for control programmes to have optimal impact (e.g. transmission-blocking vaccines versus those that decrease the probability of infection) [64].

#### 5.3.2. Model refinements

Although the model we present here captures a greater degree of host immune responses than the standard models, it still represents a simplification of the full scope of possible host–pathogen interactions and thus may be insufficient to model diseases with more complex immune dynamics. Moreover, our model does not account for the changing genetic structures of different pathogens. For example, infection with measles results in strong lifelong cross-protection against all virus strains [23], and so new invading strains are unlikely to change the susceptible profile of the population. Exposure against influenza, however, only generates partial cross-protection against other circulating strains within the same subtype [41]. In turn, there is strong selection pressure on the virus for rapid mutation, or antigenic drift, that increases the likelihood that previously infected individuals will become susceptible to new, invading strains [23]. The relative contribution of waning host immunity and viral evolution to the recruitment rate of susceptible individuals, and thus to the overall strength of buffering in response to demographic variation, is an interesting question for future work.

Finally, future analysis could also explore the impact of immigration on buffering dynamics and incorporate age structure into the refined model. Age structure is crudely incorporated in the current framework as individuals must first pass through the primary classes after birth, before entering the subsequent classes. An interesting avenue for future work would be to explicitly include age structure, in particular when the partial immunity parameters defined here vary significantly with age, as has been suggested for RSV [14,36,65]. Changes in the immigration rate are expected to have a different impact on disease dynamics from changes in the birth rate depending on the relationships between age, immunity and transmission rates. For example, one reason for increased incidence of typhoid fever in Kathmandu, Nepal, may be the immigration of susceptible migrant male works from rural areas [66]. Conversely, immigration has been found to have a negligible effect on measles dynamics once the initial epidemic growth phase has started [67], and so may be less important for acute childhood infections. Since the overall importance of immigration is likely to depend on the age demographics of both the migrant and source populations, this could be integrated into future models with explicit age structure as discussed above.

## 6. Conclusion

In this work, we have presented a general framework to model the dynamics of partially immunizing infections and shown that with just a few additional parameters we can capture a range of responses to demographic variation that would not be possible with the standard SIR and SIRS models more commonly used in the literature. Empirical estimates of the extra parameters that describe the dynamics of partial immunity have been explored for a number of diseases, such as rotavirus and RSV, through both experimental and modelling studies. For other pathogens, experimental studies or inferential methods applying this modified framework to time-series incidence data may provide insight into these relevant epidemiological parameters. This could, in turn, allow the use of models that can more accurately capture the population-level impact of the interplay between immunity and host demographics that we have described here.

## Supplementary Material

Supplementary Material
