# Supplementary material for: Demographic buffering: titrating the effects of birth rate and imperfect immunity on epidemic dynamics
Source: J R Soc Interface. 2015 Mar 6;12(104):20141245. doi: 10.1098/rsif.2014.1245 (PMC4345488; doi:10.1098/rsif.2014.1245)
Supplement: Supplementary Material [file rsif20141245supp1.pdf]

DEMOGRAPHIC BUFFERING: TITRATING THE EFFECTS OF BIRTH RATE AND IMPERFECT IMMUNITY ON EPIDEMIC DYNAMICS

Sinead E. Morris[1*], Virginia E. Pitzer[2,3], Cécile Viboud[3], C. Jessica E. Metcalf[1,3], Ottar N. Bjørnstad[3,4], Bryan T. Grenfell[1,3].

[1] Department of Ecology and Evolutionary Biology, Princeton University, Princeton, New Jersey, USA.
[2] Department of Epidemiology of Microbial Diseases, Yale School of Public Health, New Haven, Connecticut, USA.
[3] Fogarty International Center, National Institutes of Health, Bethesda, Maryland, USA.
[4] Center for Infectious Disease Dynamics, Departments of Entomology and Biology, Pennsylvania State University, University Park, Pennsylvania, USA
* Corresponding author: S. E. Morris; semorris@princeton.edu

Supplementary Material

S1. SIRS phase portrait — 2

S2. Methods — 3

S2.1. Model parameters — 3

S2.2. Pathogen-specific immunological parameters — 3

S2.3. Quantifying the extent of buffering — 3

S3. Measuring changes in disease incidence — 5

S3.1. Continuously changing relative infectiousness — 5

S3.2. Changing population size — 6

S3.3. Incorporating reduced reporting of subsequent cases — 8

S3.4. Variations in R_0 — 9

S4. Measuring changes in timing — 10

S4.1. Reduced reporting of subsequent cases — 10

S4.2. Timing of epidemic peaks as an alternative measurement — 11

S4.3. Changing population size — 11

References — 16

S1. SIRS phase portrait

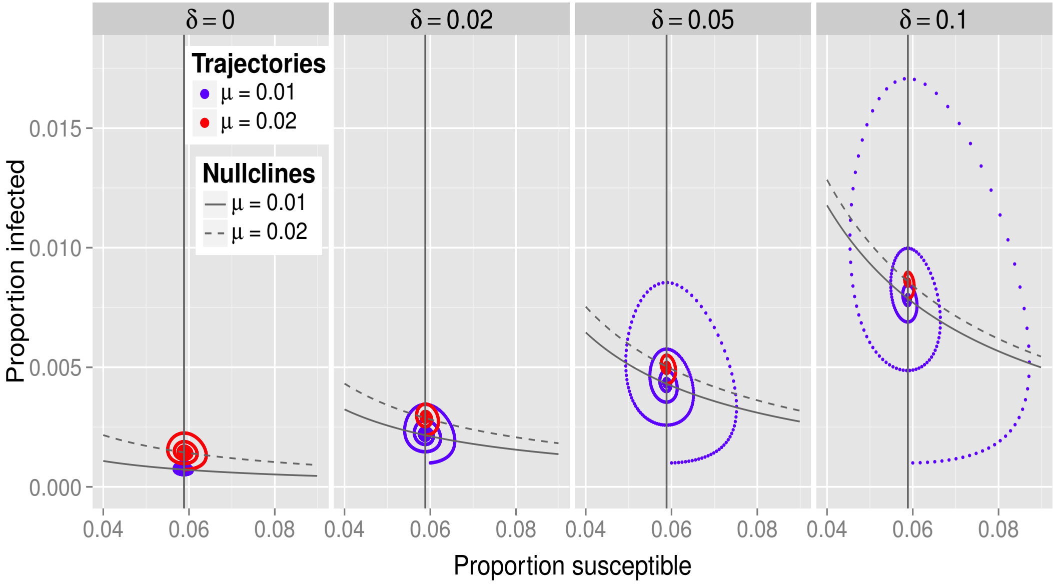

Figure S1. Phase portrait of the dynamics of the unforced SIRS model for different rates of loss of immunity (δ). Simulations were run with an initial birth rate of $\mu = 0.01$ yrs^{-1} (blue points) until equilibrium was reached. Then the birth rate was increased to 0.02 yrs^{-1} and the simulations were continued (red points) until the new equilibrium was reached. Nullclines for the susceptibles (given by $dS/dt = 0$) and infecteds ($dI/dt = 0$) are plotted in grey (vertical lines are the I-nullclines and curves are the S-nullclines) with the solid lines representing $\mu = 0.01$ yrs^{-1} and the dashed lines $\mu = 0.02$ yrs^{-1}. The solid and dashed I-nullclines overlap. Each equilibrium is the point at which the respective S- and I-nullclines intersect. The reproductive number, R_0, is 17, and all other parameter values for the SIRS model are as defined in Table 1 (main document).

S2. Methods

S2.1. **Model parameters.** Parameter values for all simulations, unless stated otherwise, are given in Table 1 (main document). These parameters, with the incorporation of seasonal forcing via a simple sinusoidal function, were chosen to generate yearly epidemic cycles that are qualitatively similar to dynamics that have been recorded for many childhood infections, including measles, rotavirus and RSV [1–3]. Moreover, these annual oscillations allow changes in disease incidence and epidemic timing resulting from demographic variation to be tracked on a yearly basis through the course of the simulations.

Turnover birth rates ranging between 0.01–0.02 yrs^{-1} are consistent with those reported in developed countries over the last 50 years [4]. For example, birth rates in the United Kingdom fell from 0.018 to 0.012 yrs^{-1} over a period of 20 years following the post-World War II 'baby boom' [4, 5] and state-level birth rates in the United States in 2012 ranged from 0.0094–0.018 yrs^{-1} [6]. For all simulations in this study, the baseline birth rate was 0.01 yrs^{-1}. Once equilibrium was reached with this constant value, the extent of buffering of disease incidence in response to demographic changes was investigated by introducing a 'birth pulse' into the population dynamics (Figure 1, main document, top left panel). To further investigate the effect of demographic changes on the timing of epidemics, the birth rate was set to be monotonically increasing (Figure 1, top left panel) so that both the transient and permanent effects on epidemic timing could be compared across models. Simulations were stopped once the system had regained equilibrium following the change in birth rate.

S2.2. **Pathogen-specific immunological parameters.** Table S1 gives the parameters governing partial immunity for the different infections considered in the main text.

Table S1. Approximate values, obtained from a range of experimental and modeling studies, for the relevant immunological parameters of specific pathogens highlighted in the text.

Pathogen	Duration of immunity ($1/\delta$)	Relative infectiousness of subsequent infections (α)	Relative susceptibility to subsequent infections (ϵ)	References
Measles	Lifelong	- *	- *	[7]
Pertussis	10 yrs	0.5	0.25	[8–10]
Influenza	5 yrs	0.5	0.45	[11]
Rotavirus	9 months	0.1	0.35	[12–15]
RSV	6 months	0.25	0.45	[16–19]

* Relative infectiousness and susceptibility to reinfection do not apply in the case of measles since immunity to the pathogen is lifelong. However, for comparison purposes in Figures 3, S3 and S4 we represent measles in the range where $\alpha = 1$ and $\epsilon = 0$.

S2.3. **Quantifying the extent of buffering.** The slope of the regression line of change in birth rate against change in disease incidence gives a measure of the extent of buffering in response to demographic variation (discussed further in Section 2.2, main document). Values close to 1 correspond to low buffering since there is an almost proportional change in incidence in response to the change in birth rate, whereas values near 0 correspond to high buffering since there is almost no change. This measure is used to compare buffering levels between the standard and modified models and across different parameter ranges.

Two measures were used to quantify the change in timing of the yearly epidemic oscillations. Firstly, the mean timing, or center of mass, of each annual cycle was calculated as

$$\frac{\sum\limits_{d \in [-182,182]} dI_d}{\sum\limits_{d \in [-182,182]} I_d},$$

where d is an index for each day of the year and I_d is the total number of infections on day d. The midpoint of the year was chosen to coincide with the timing of peak transmission, such that $d = (1, 2, ..., 182)$ represented days 1, 2,..., 182 and $d = (-182, -181, ..., 0)$ represented days 183, 184,..., 365. Secondly, the timing of the peak of each annual epidemic was calculated as the day at which the maximum turning point of the cycle occurred. For each measure, the change in epidemic timing was taken as the difference between the calculated value for the final year of the simulation and the year immediately prior to the change in birth rate. Negative values therefore indicate that the demographic changes caused epidemics to occur earlier. All analyses were performed using the R 3.0.3 package 'deSolve' [20, 21].

S3. Measuring changes in disease incidence

S3.1. Continuously changing relative infectiousness.

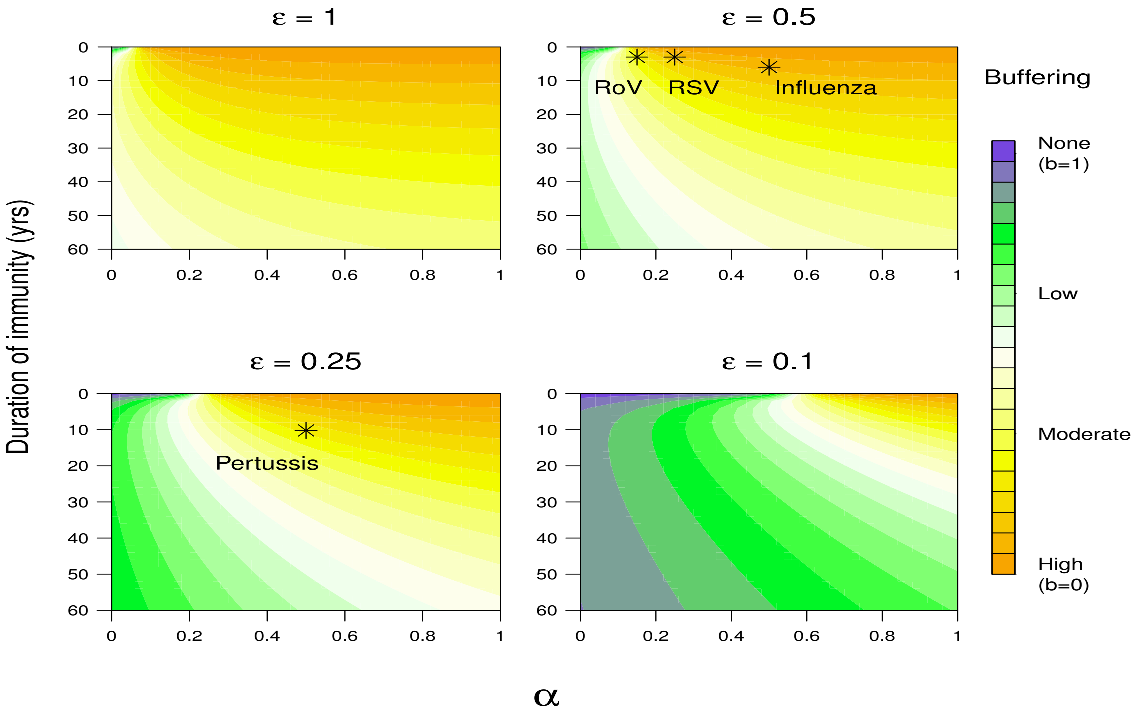

Figure S2. Using the slope of the regression line of birth rate against disease incidence to measure the extent of buffering in response to a change in birth rate. Slopes close to 0 correspond to high buffering and slopes near 1 correspond to low or no buffering. The duration of immunity is $1/\delta$, where $\delta \in [0, 1]$, and ϵ and α are the relative susceptibility and infectiousness of subsequent infections, respectively. The birth rate function is as shown in the left panel of Figure 1, the reproductive number, R_0, is 17, and all other parameters are as defined in Table 1. Points represent regions of expected buffering levels for the different labeled diseases ('RoV' stands for rotavirus) and pathogen-specific parameters are as defined in Table S1.

S3.2. **Changing population size.**

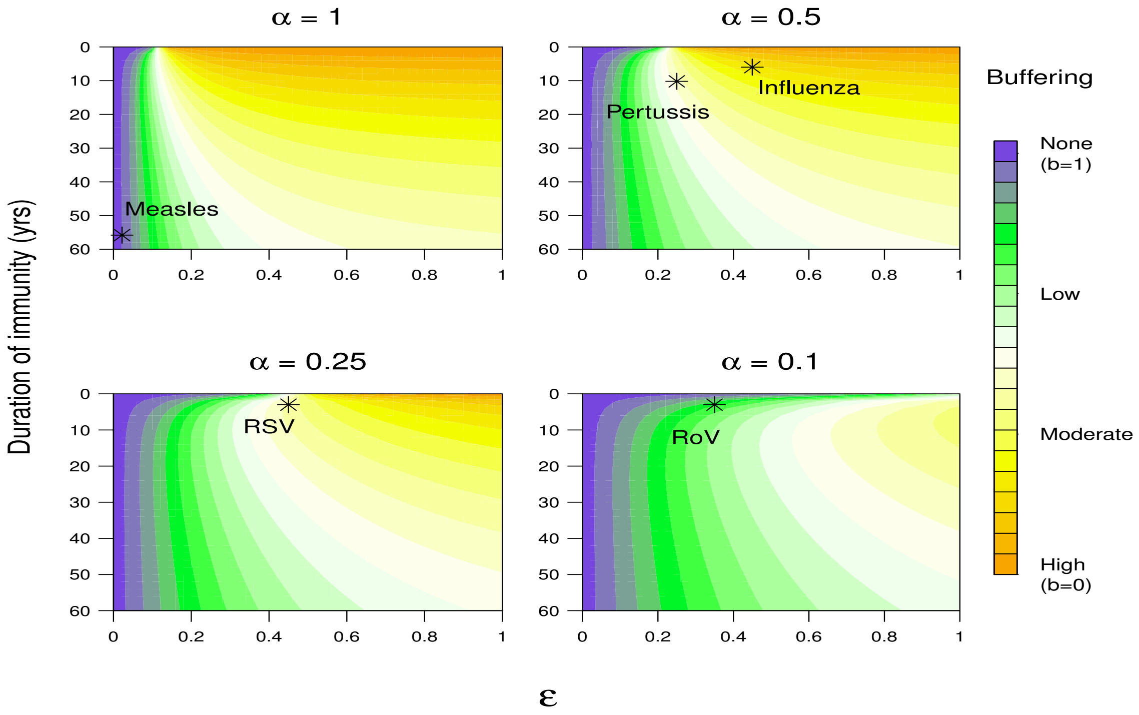

Figure S3. Frequency-dependent model with changing population size: using the slope of the regression line of change in birth rate against disease incidence to measure the extent of buffering. The birth rate ranges from 0.01 yrs^{-1} to 0.02 yrs^{-1}, the death rate is 1/75 yrs^{-1}, and ϵ and α are the relative susceptibility and infectiousness of subsequent infections, respectively. The reproductive number, R_0, is 17, and all other parameter values are as defined in Table 1. Points represent regions of expected buffering levels for the different labeled diseases ('RoV' stands for rotavirus) and pathogen-specific parameters are as defined in Table S1.

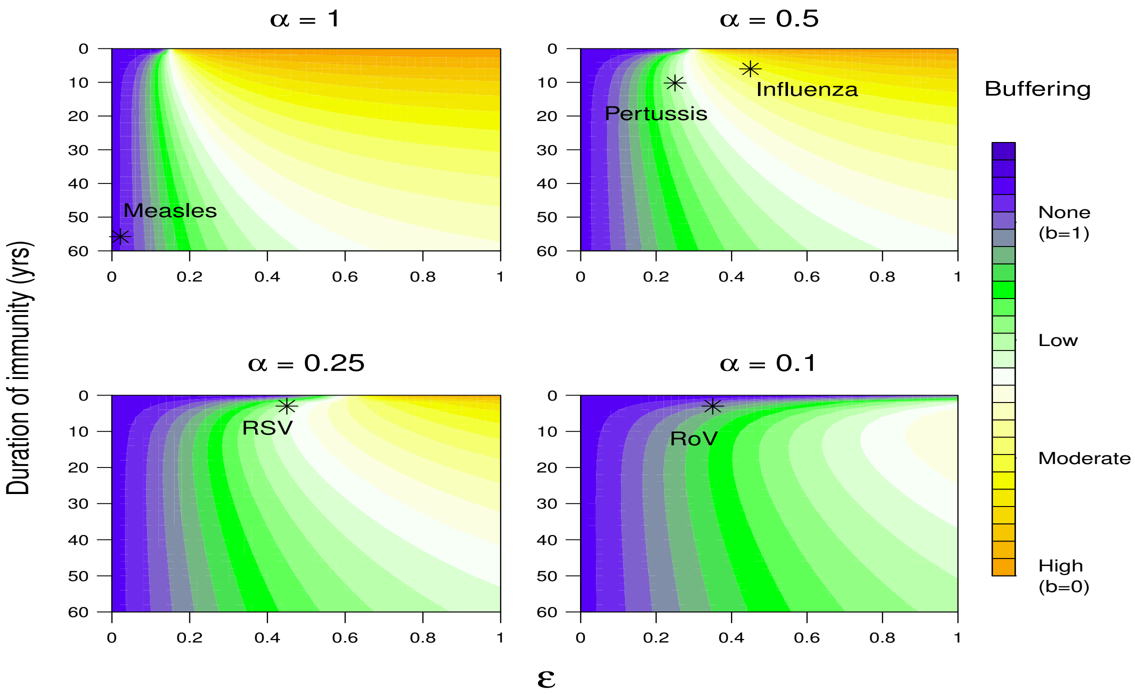

Figure S4. Density-dependent model with changing population size: using the slope of the regression line of change in birth rate against disease incidence to measure the extent of buffering. The birth rate ranges from 0.01 yrs^{-1} to 0.02 yrs^{-1}, the death rate is 1/75 yrs^{-1}, and ϵ and α are the relative susceptibility and infectiousness of subsequent infections, respectively. The reproductive number, R_0, is 17, and all other parameter values are as defined in Table 1. Points represent regions of expected buffering levels for the different labeled diseases ('RoV' stands for rotavirus) and pathogen-specific parameters are as defined in Table S1.

S3.3. **Incorporating reduced reporting of subsequent cases.**

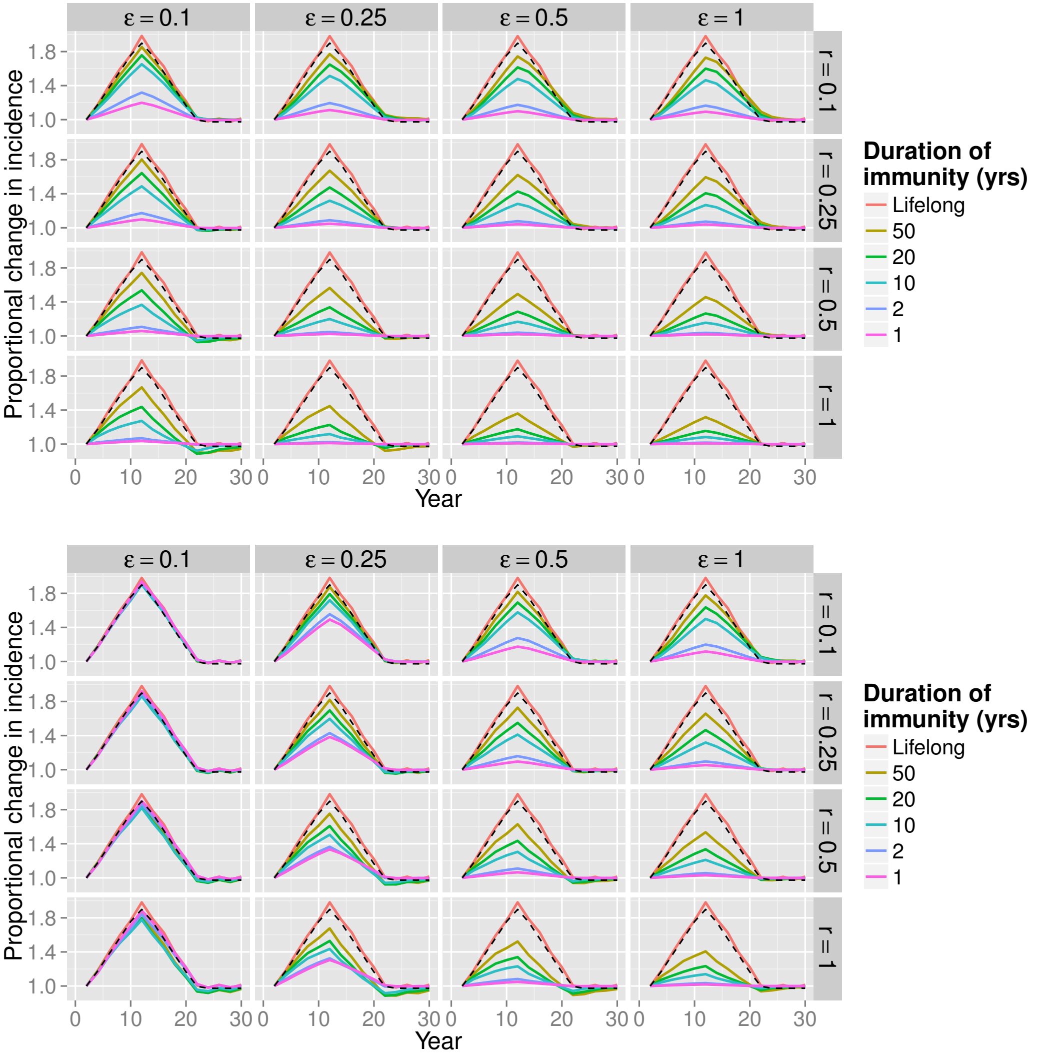

Figure S5. Plot of the proportional change in $I_P + rI_S$, where r is the proportion of subsequent cases that are reported. The dashed black line represents the change in birth rate, the duration of immunity is calculated as $1/\delta$, and ϵ is the relative susceptibility of subsequent infections. The relative infectiousness of subsequent infections, α, is set to 1 (top) and 0.25 (bottom), and R_0=17. The birth rate function is as shown in the left panel of Figure 1, and all other parameters are as defined in Table 1.

S3.4. **Variations in R_0.**

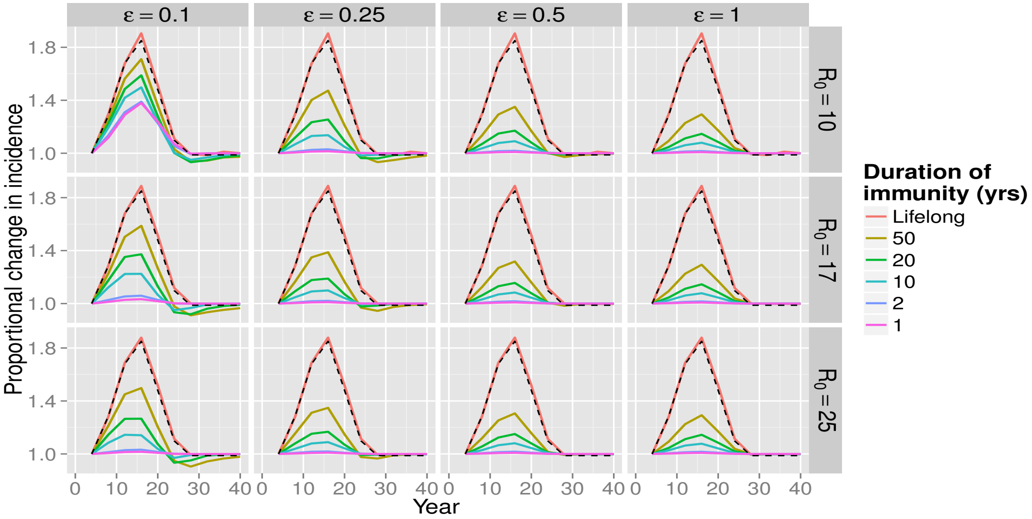

Figure S6. Plot of the proportional change in incidence induced by a change in birth rate for different values of the reduced susceptibility to subsequent infections, ϵ, and R_0. The relative infectiousness of reinfections, α, and the proportion of subsequent cases that are reported, r, are both equal to 1. The birth rate function is as shown in the left panel of Figure 1, and all other parameters are as defined in Table 1.

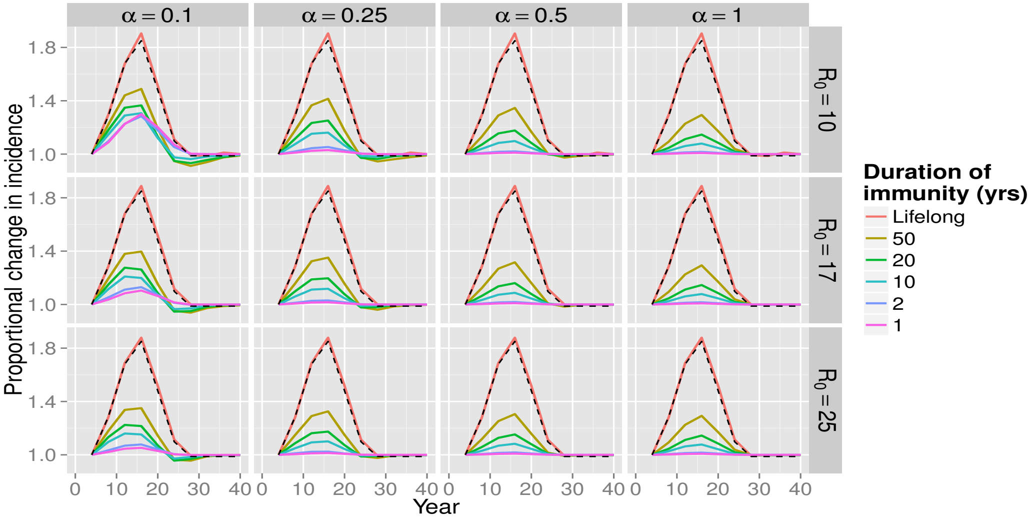

Figure S7. Plot of the proportional change in incidence induced by a change in birth rate for different values of the reduced infectiousness of subsequent infections, α, and R_0. The relative susceptibility to reinfections, ϵ, and the proportion of subsequent cases that are reported, r, are both equal to 1. The birth rate function is as shown in the left panel of Figure 1, and all other parameters are as defined in Table 1.

S4.1. **Reduced reporting of subsequent cases.**

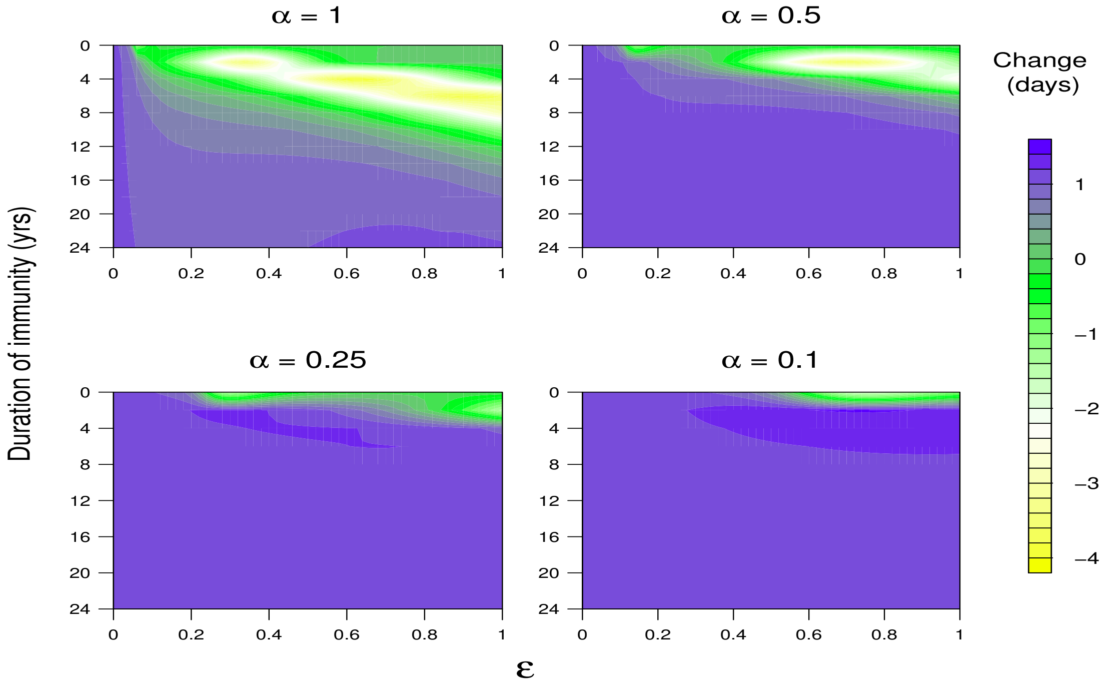

Figure S8. Change in mean timing (days) of epidemic oscillations induced by an increase in birth rate. The duration of immunity is $1/\delta$, where $\delta \in [0, 1]$, and ϵ and α are the relative susceptibility and infectiousness of subsequent infections, respectively. The reproductive number, R_0, is 17, and the proportion of subsequent cases that are reported, r, is 0.25. The birth rate function is as shown in the left panel of Figure 1, and all other parameters are as defined in Table 1.

S4.2. **Timing of epidemic peaks as an alternative measurement.**

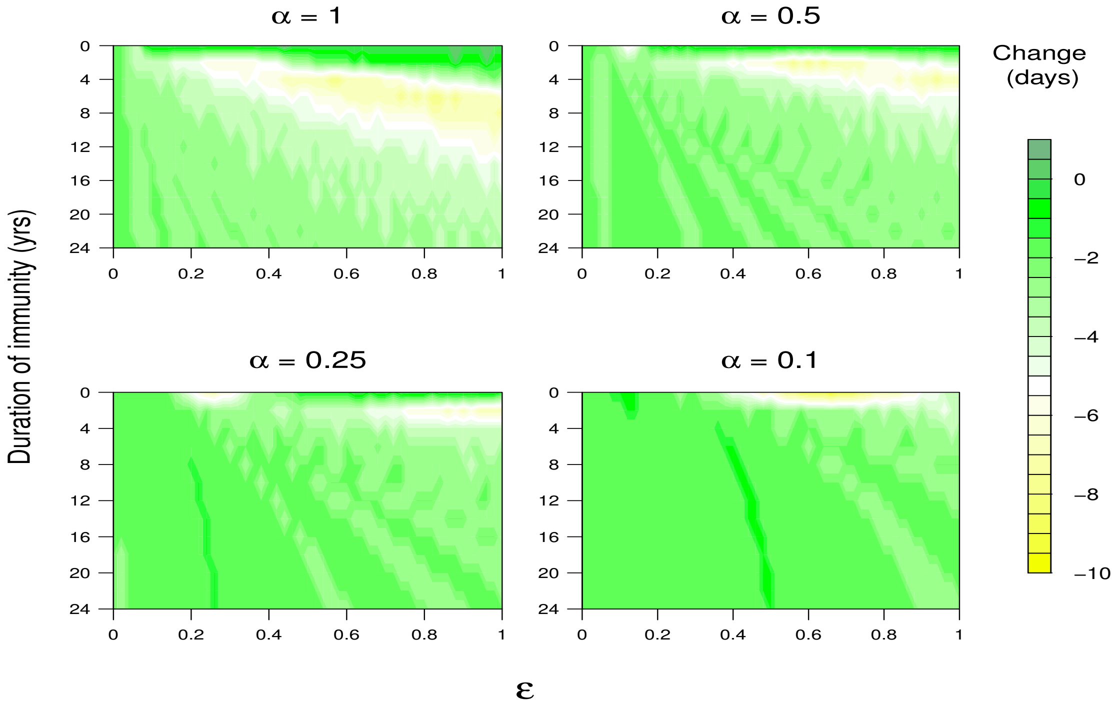

Figure S9. Change in peak timing (days) of epidemic oscillations induced by an increase in birth rate. The peak is measured as the maximum turning point in the total number of infected individuals during each yearly cycle. The duration of immunity is $1/\delta$, where $\delta \in [0, 1]$, and ϵ and α are the relative susceptibility and infectiousness of subsequent infections, respectively. The reproductive number, R_0, is 17, and the proportion of subsequent cases that are reported, r, is 1. The birth rate function is as shown in the left panel of Figure 1, and all other parameters are as defined in Table 1.

S4.3. **Changing population size.** Quantifying the effects of demographic variation on the change in timing for the models with non-constant population size is more challenging since the population size changes in response to the increase in births in such a way as to ensure that the long-term turnover rate remains constant and the system maintains equilibrium. Considering the transient changes induced by the increase in birth rate, however, reveals significant differences between the frequency and density-dependent dynamics. In the frequency-dependent simulations, the mean and peak timings of the epidemic oscillations tend to return back to the original values once the birth increase has come to an end (Figures S13 and S12), whereas in the density-dependent simulations they do not (Figures S11 and S10). Moreover, in the density-dependent simulations there are greater changes in timing during the transient period than compared to the frequency-dependent simulations. This greater sensitivity to demographic changes is expected with the density-dependent model since transmission rates scale with population size.

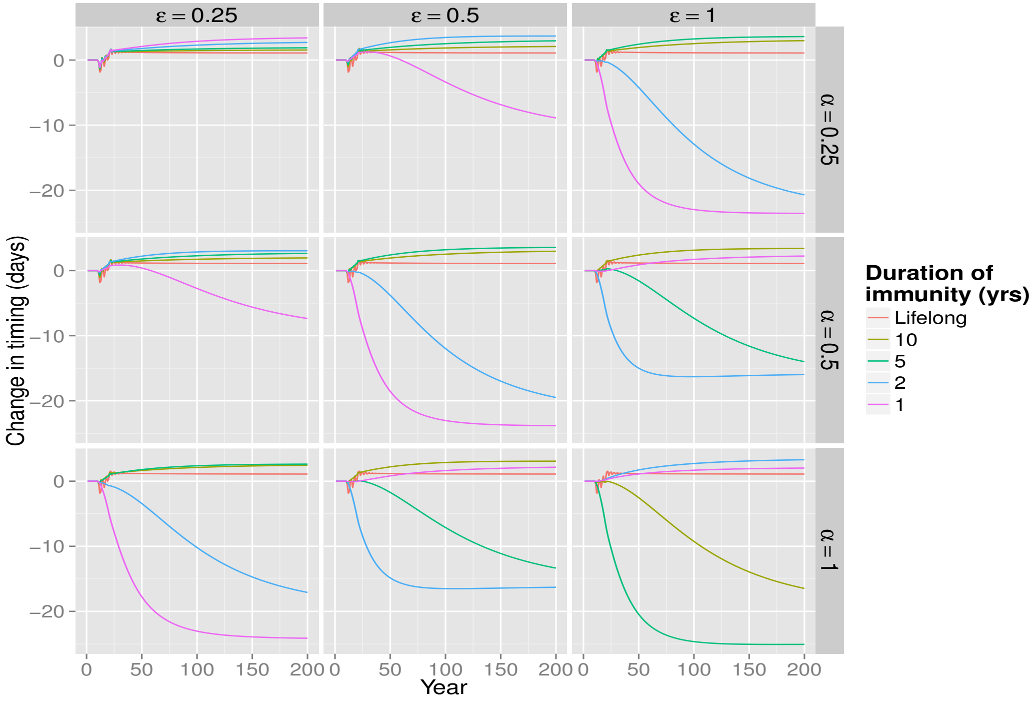

Figure S10. Density-dependent model with changing population: plot of how the mean timing of the epidemics changes during and after the period of an increase in number of births. The birth rate ranges from 0.01 yrs^{-1} to 0.02 yrs^{-1} and the death rate is 1/75 yrs^{-1}. The change in time refers to the day of the mean timing relative to that measured at the beginning of the simulation. The reproductive number, R_0, is 17, the proportion of subsequent cases reported, r, is 1, and all other parameters are as defined in Table 1.

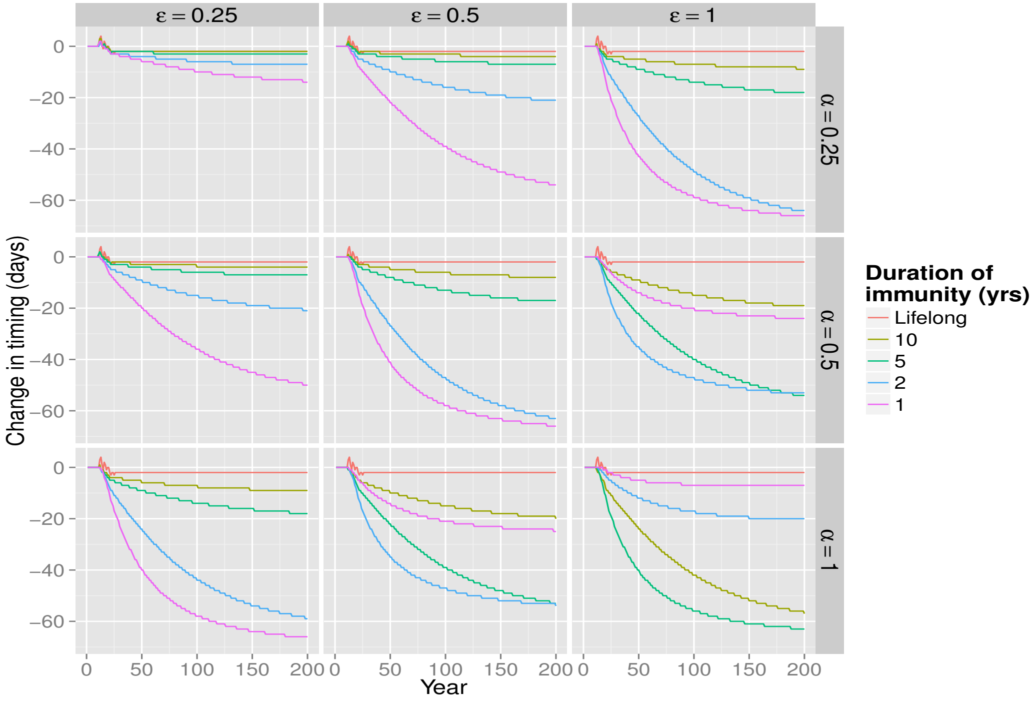

Figure S11. Density-dependent model with changing population: plot of how the timing of the epidemic peak changes during and after the period of an increase in number of births. The birth rate ranges from 0.01 yrs^{-1} to 0.02 yrs^{-1} and the death rate is 1/75 yrs^{-1}. The change in timing refers to the day of the peak timing relative to that measured at the beginning of the simulation. The reproductive number, R_0, is 17, the proportion of subsequent cases reported, r, is 1, and all other parameters are as defined in Table 1.

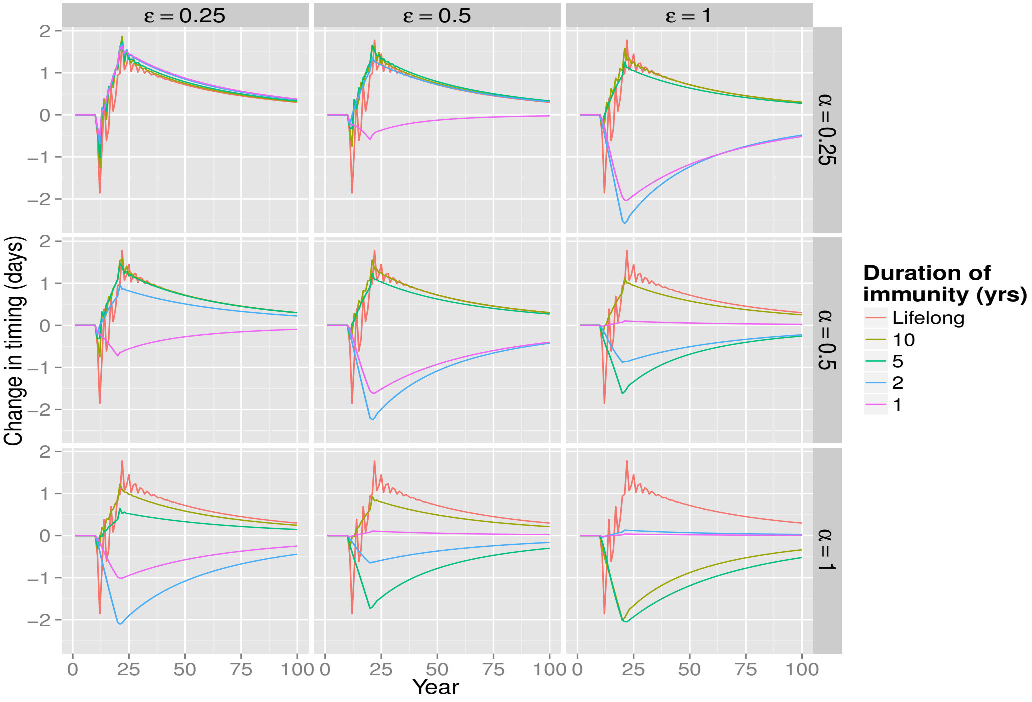

Figure S12. Frequency-dependent model with changing population: plot of how the mean timing of the epidemics changes during and after the period of an increase in number of births. The birth rate ranges from 0.01 yrs^{-1} to 0.02 yrs^{-1} and the death rate is 1/75 yrs^{-1}. The change in time refers to the day of the mean timing relative to that measured at the beginning of the simulation. The reproductive number, R_0, is 17, the proportion of subsequent cases reported, r, is 1, and all other parameters are as defined in Table 1.

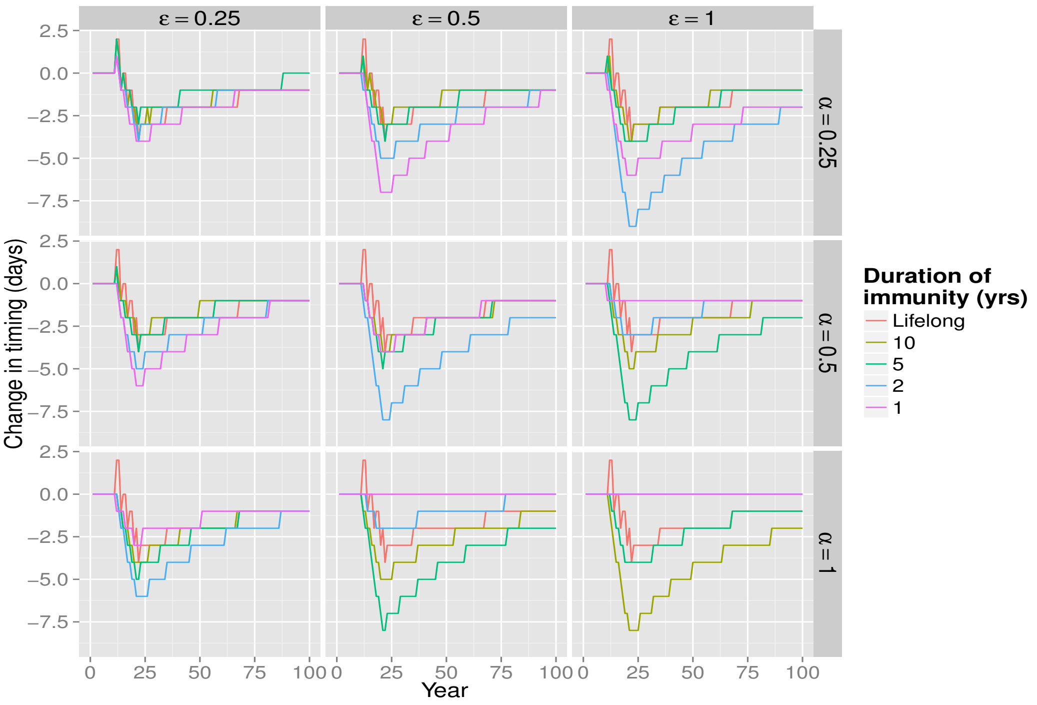

Figure S13. Frequency-dependent model with changing population: plot of how the timing of the epidemic peak changes during and after the period of an increase in number of births. The birth rate ranges from 0.01 yrs^{-1} to 0.02 yrs^{-1} and the death rate is 1/75 yrs^{-1}. The change in time refers to the day of the peak timing relative to that measured at the beginning of the simulation. The reproductive number, R_0, is 17, the proportion of subsequent cases reported, r, is 1, and all other parameters are as defined in Table 1.

References

[1] Grenfell, B. T., Bjørnstad, O. N., and Kappey, J. 2001. Travelling waves and spatial hierarchies in measles epidemics. *Nature.* **414**, 716–723. (DOI: 10.1038/414716a)

[2] LeBaron, C. W., Lew, J., Glass, R. I., Weber, J. M., and Ruiz-Palacios, G. M. 1990. Annual rotavirus epidemic patterns in North America: results of a 5-year retrospective survey of 88 centers in Canada, Mexico, and the United States. *JAMA.* **264**, 983–988. (DOI: 10.1001/jama.1990.03450080069033)

[3] Noyola, D. E. and Mandeville, P. B. 2008. Effect of climatological factors on respiratory syncytial virus epidemics. *Epidemiology and Infection.* **136**, 1328–1332. (DOI: 10.1017/S0950268807000143)

[4] United Nations Population Division: Department of Economic and Social Affairs. World Population Prospects: The 2012 Revision. 2012. URL: http://esa.un.org/wpp/Excel-Data/fertility.htm (Accessed 08/21/2014).

[5] Grenfell, B. T., Bjørnstad, O. N., and Finkenstädt, B. F. 2002. Dynamics of measles epidemics: scaling noise, determinism, and predictability with the TSIR model. *Ecological Monographs.* **72**, 185–202. (DOI: 10.1890/0012-9615(2002)072[0185:DOMESN]2.0.CO;2)

[6] Martin, J. A., Hamilton, B. E., Osterman, M. J. K., Curtin, S. C., and Mathews, T. J. National Vital Statistics Report, Births: Final Data for 2012. 2013. URL: http://www.cdc.gov/nchs/data/nvsr/nvsr62/nvsr62_09.pdf (Accessed 08/21/2014).

[7] Anderson, R. M. and May, R. M. 1992. *Infectious Diseases of Humans: Dynamics and Control.* Oxford: Oxford Science Publications.

[8] Wendelboe, A. M., Van Rie, A., Salmaso, S., and Englund, J. A. 2005. Duration of immunity against pertussis after natural infection or vaccination. *The Pediatric Infectious Disease Journal.* **24**, S58–S61. (DOI: 10.1097/01.inf.0000160914.59160.41)

[9] Versteegh, F. G. A., Mertens, P. L. J. M., De Melker, H. E., Roord, J. J., Schellekens, J. F. P., and Teunis, P. F. M. 2005. Age-specific long-term course of IgG antibodies to pertussis toxin after symptomatic infection with Bordetella pertussis. *Epidemiology and Infection.* **133**, 737–748. (DOI: 10.1017/S0950268805003833)

[10] Águas, R., Gonçalves, G., and Gomes, M. G. M. 2006. Pertussis: increasing disease as a consequence of reducing transmission. *The Lancet infectious diseases.* **6**, 112–117. (DOI: 10.1016/S1473-3099(06)70384-X)

[11] Davies, J. R., Grilli, E. A., and Smith, A. J. 1986. Infection with influenza A H1N1: 2. The effect of past experience on natural challenge. *Journal of hygiene.* **96**, 345–352. (DOI: 10.1017/S0022172400066092)

[12] Velázquez, F. R., Matson, D. O., Calva, J. J., Guerrero, M. L., Carter-Campbell, A. L. M. S., Glass, R. I., Estes, M. K., Pickering, L. K., and Ruiz-Palacios, G. M. 1996. Rotavirus infection in infants as protection against subsequent infections. *New England Journal of Medicine.* **335**, 1022–1028. (DOI: 10.1056/NEJM199610033351404)

[13] Chiba, S., Nakata, S., Urasawa, T., Urasawa, S., Yokoyama, T., Morita, Y., Taniguchi, K., and Nakao, T. 1986. Protective effect of naturally acquired homotypic and heterotypic rotavirus antibodies. *The Lancet.* **328**, 417–421. (DOI: 10.1016/S0140-6736(86)92133-1)

[14] Koopman, J. S. and Monto, A. S. 1989. The Tecumseh study XV: Rotavirus infection and pathogenicity. *American Journal of Epidemiology.* **130**, 750–759.

[15] Pitzer, V. E., Viboud, C., Simonsen, L., Steiner, C., Panozzo, C. A., Alonso, W. J., Miller, M. A., Glass, R. I., Glasser, J. W., Parashar, U. D., and Grenfell, B. T. 2009. Demographic Variability, Vaccination, and the Spatiotemporal Dynamics of Rotavirus Epidemics. *Science.* **325**, 290–294. (DOI: 10.1126/science.1172330)

[16] Henderson, F. W., Collier, A. M., Clyde Jr, W. A., and Denny, F. W. 1979. Respiratory-syncytial-virus infections, reinfections and immunity: a prospective, longitudinal study in young children. *New England Journal of Medicine.* **300**, 530–534. (DOI: 10.1056/NEJM197903083001004)

[17] Nokes, D. J., Ngama, M., Bett A.and Abwao, J., Munywoki, P., English, M., Scott, J. A. G., and Cane P. A .and Medley, G. F. 2009. Incidence and severity of respiratory syncytial virus pneumonia in rural Kenyan children identified through hospital surveillance. *Clinical infectious diseases.* **49**, 1341–1349. (DOI: 10.1086/606055)

[18] Hall, C. B., Walsh, E. E., Long, C. E., and Schnabel, K. C. 1991. Immunity to and frequency of reinfection with respiratory syncytial virus. *Journal of Infectious Diseases.* **163**, 693–698. (DOI: 10.1093/infdis/163.4.693)

[19] White, L. J., Mandl, J. N., Gomes, M. G. M., Bodley-Tickell, A. T., Cane, P. A., Perez-Brena, P., Aguilar, J. C., Siqueira, M. M., Portes, S. A., Straliotto, S. M., et al. 2007. Understanding the transmission dynamics of respiratory syncytial virus using multiple time series and nested models. *Mathematical Biosciences.* **209**, 222–239. (DOI: 10.1016/j.mbs.2006.08.018)

[20] R Core Team. *R: A Language and Environment for Statistical Computing.* R Foundation for Statistical Computing. Vienna, Austria, 2014. URL: http://www.R-project.org/.

[21] Soetaert, K., Petzoldt, T., and Setzer, W. R. 2010. Solving Differential Equations in R: Package deSolve. *Journal of Statistical Software.* **33**, 1–25.
